# Extended reality for mapping perforator-based flaps in breast reconstruction: a systematic review and meta-analysis

**DOI:** 10.1016/j.jpra.2025.02.011

**Published:** 2025-02-27

**Authors:** Hester Lacey, Anas Khan, Baljit Dheansa

**Affiliations:** a University Hospitals Sussex NHS Foundation Trust, Eastern Rd, Brighton and Hove, Brighton, BN2 5BE, UK; b Department of Plastic Surgery, Queen Victoria Hospital, Holtye Rd, East Grinstead, RH19 3DZ, UK

**Keywords:** Extended reality, Perforator flaps, Breast reconstruction

## Abstract

**Introduction:**

Extended reality technologies including augmented reality (AR) and virtual reality (VR) can be used in surgical settings for surgical planning, perioperative visualisation of patient anatomy and simulation of operative steps. This study aimed to ascertain the role of extended reality in perforator-based breast reconstruction.

**Methods:**

A systematic search of the literature was performed. Screening was conducted independently by two reviewers, with conflicts resolved through consensus.

**Results:**

In total, 4957 articles were identified of which 10 were included, comprising 229 flaps. Overall, localisation of perforator emergence with AR and VR were 4 mm (95% confidence interval [CI] 3.2-4.9) and 14 mm (95% CI 5.5-22.5), respectively, from the correct intraoperatively identified location. Two studies reported a significant reduction in harvesting time (88 min for VR and 19 min for AR). The pooled mean image processing time from four studies was 39±47 min. Preoperative VR was associated with shorter operative times than conventional Doppler ultrasound (478±56.94 vs 606.29 ± 81.94 min, P<0.05). Use of a simulated environment for mapping perforators appeared to reduce complications (wound breakdown, flap revision, flap loss, infection); however, this failed to reach statistical significance (odds ratio 0.6; 95% CI 0.3-1.3; P=0.20).

**Conclusions:**

This study suggests that AR and VR offer limited benefit in improving accuracy of perforator identification; however, they may reduce flap harvesting and total operative time. Key limitations include heterogeneity and quality of the included studies. With larger sample sizes and higher quality evidence, definitive benefits and longitudinal outcomes relating to use of extended reality in perforator-based breast reconstruction may be established.

## Introduction

### Rationale

**Figure 1:**
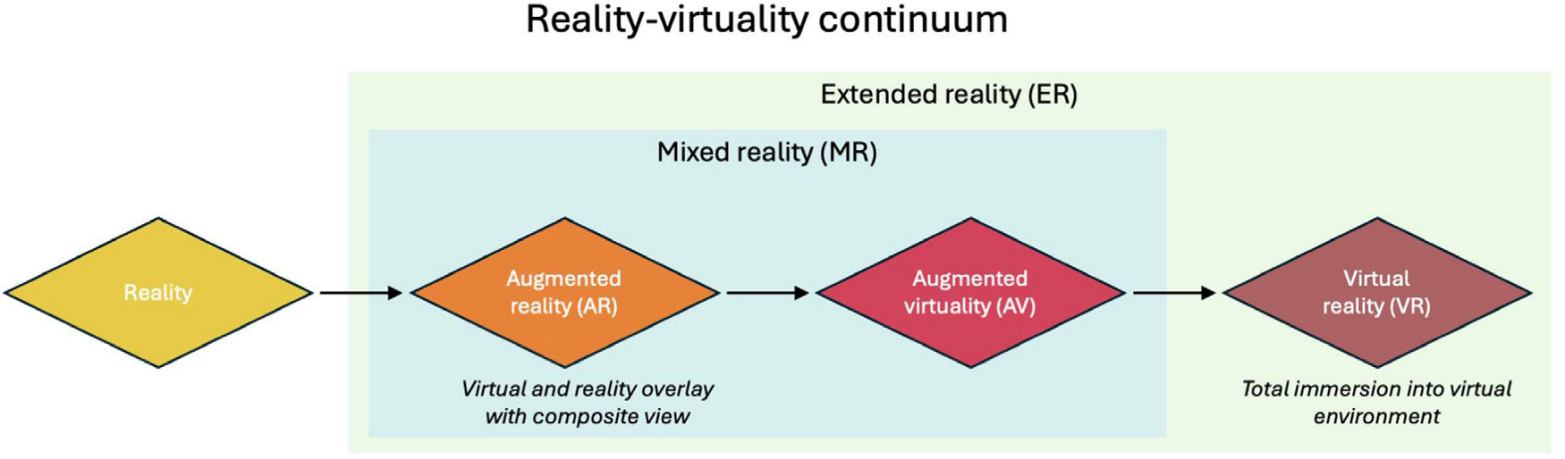
Reality–virtuality continuum.

Extended reality describes technologies that merge reality and computer-generated environments into a reality–virtuality continuum, with the overlay of visual, audio and haptic sensory inputs integrated with real-life environments in real time (Figure 1).^1^^,^^2^

Extended reality includes augmented reality (AR), augmented virtuality and virtual reality (VR), each offering various levels of immersion into the virtual world with real-life sensory integration and interactive overlay.^1^ VR describes simulated 3D environments that immerse the user in the virtual world with the ability to move and interact with stimuli in real time.^2^ This is achieved with a VR headset, which uses pose and head tracking of the operator's movements to personalise the virtual experience and increase sensory immersion or a projector, mobile or desktop-based VR system.^3^ Popular applications of VR include educational and entertainment contexts, including simulation-based learning and computer games that incorporate game player interaction.^4^^,^^5^

AR is a form of extended reality involving the interactive overlap of computer-generated 3D content onto real-life environments.^6^ Differing from VR by the combination of partially generated reality layered onto the real world, AR was initially popularised through integration into marketing campaigns.^7^ From the development of the first AR technology, the complexity and scope of AR has expanded dramatically, with AR technology now adopted into mainstream culture through AR mobile apps and video games.^8^ Visual projection of computer-generated content can be achieved with use of headsets, glasses or smartphones, with overlay of additional sensory modalities including haptic, auditory and olfactory content to offer a multimodal, immersive AR experience seamlessly integrated with the real world.^9^

With advances in data and computer processing, motion tracking and projection technologies, extended and mixed reality modalities have increasing scope for integration into industry and workplace settings.^10^ In surgical settings, the use of extended reality to aid perioperative visualisation, planning and simulation of operative steps or to complete procedures is being increasingly explored.^4^ VR offers a risk-free opportunity to interact with the operative environment with significant educational benefits, enabling trainees to practice operative steps before real-life operating theatre experience.^9^ This reduces patient risk and facilitates improved operative skills early in training, with unlimited opportunities to practice surgical skills and perfect techniques.^11^ AR is typically used in real time in the operative environment, with anatomical projection during surgery aiding the visualisation and comprehensive assessment of patient anatomy and pathology.^6^ Effective AR integration has the potential to enhance surgical precision, aid preoperative planning, optimise intraoperative procedure steps and reduce complications and operative times.^12^

In plastic and reconstructive surgery, effective intraoperative identification of detailed vascular anatomy is an essential step of both pedicled and free tissue transfer.^13^ In breast reconstruction, perforator flaps, including the deep inferior epigastric artery perforator (DIEP), transrectus abdominus myocutaneous (TRAM) and less commonly, thigh-based perforator flaps, are a common and effective reconstructive option.^14^ Perforator flaps offer improved cosmetic postoperative results compared to implant reconstruction; however, successful perforator flap dissection is technically challenging and can be time-consuming.^15^ Three-dimensional extended reality, such as AR and VR, offers a novel method of perioperative anatomical navigation with significant potential for aiding these technologically challenging procedures.^8^ In breast reconstruction, VR may be used preoperatively with computed tomography (CT) and magnetic resonance (MR) imaging to model vascular anatomy, practice intraoperative anatomical navigation and aid preoperative planning, navigate technical and anatomical challenges prior to entering the operating room and aid determination of the optimal surgical approach.^16^ In recent years, projection-based AR systems have been used perioperatively to guide intraoperative perforator identification with direct overlay of preoperatively imaged vascular anatomy onto the patient before or during surgery, facilitating accuracy, expediting intraoperative perforator identification and reducing error rates and operating times, with associated benefits for patient outcomes.^12^

Definitive evidence for the benefits and potential of extended reality modalities including VR and AR in aiding intraoperative perforator identification for breast reconstruction remains limited by the novel nature of technology and its recent introduction into surgical environments.

### Objectives

This study systematically reviewed the available literature and evidence relating to mixed reality in perforator-based free flap breast reconstruction. The primary outcome was accuracy of perforator identification using extended reality compared to traditional imaging technologies, with secondary outcomes including flap harvesting and total operating times. The effects on associated complications and financial cost were also assessed. The authors hypothesised that extended reality technology would improve the accuracy of perforator identification by optimising perioperative planning and reducing total operative time but would potentially increase the financial cost associated with technology integration.

## Methods

### Search strategy and information sources

A systematic search was conducted using a combination of key terms such as ‘augmented reality’, ‘virtual reality’, ‘perforator’, and ‘breast reconstruction’ and various synonyms in different combinations. These search terms were developed in collaboration with medical librarians to ensure the highest possible capture rate. The exact search terms and strategy are detailed in Supplement 1. The search was performed in seven databases: The Cochrane Library (including the CENTRAL component for randomised controlled trials), MEDLINE, EMBASE, Global Health, Web of Science, Scopus and the grey literature source Google Scholar. Additionally, the reference lists of key papers were reviewed to identify additional relevant articles. The initial search was performed in July 2024 with an interval search performed in December 2024 to ensure latest study data was captured.

### Eligibility criteria

The population, intervention, comparison and outcome (PICO) framework was used to guide paper choice. Articles were included if they reported the effects of extended reality on either preoperative or intraoperative perforator identification. Any type of perforator flap (abdominal or thigh) was included. The primary outcome was accuracy. Secondary outcomes included harvesting time, total operative time, complications, ease of use and financial cost. Primary quantitative research, including single arm and comparative studies, were included. Non-scientific reports, reviews and studies with less than five participants were excluded. The full inclusion and exclusion criteria are described in the supplementary files.

### Selection process

Title and abstract screening, as well as full-text review, were conducted independently by two reviewers (HL and AK) according to the study inclusion and exclusion criteria. Disagreements were resolved through consensus and in cases in which consensus could not be achieved, a third reviewer's opinion was sought.

### Data collection process

Data collection was performed using a customised spreadsheet on the Covidence platform, with two reviewers independently gathering data and resolving conflicts through consensus.^17^ The data extracted are not widely available.

### Data items

Extracted data included study demographic data (e.g., country, institution), study type, study aim, sample size, participants’ demographic characteristics (e.g., age, type/site of flap, flap size), technology used (e.g., type of device and software), intervention performed and outcomes (accuracy of perforator identification, perforator identification time, total operating time and complications).

### Study risk of bias assessment

Quality assessment was carried out by two authors independently using the Joanna Briggs Institute critical appraisal tools specific to the study design.^18^ A third author acted as adjudicator in the event of disagreement. Additionally, a summary of findings table was created and the quality of evidence was graded using the Grading of Recommendations Assessment, Development and Evaluation (GRADE) system, with evaluations performed using an online application (https://gdt.gradepro.org/app).

### Effect measures and synthesis methods

Data analysis was performed using R software (version 3.4.3) with the meta package.^19^ Pooled estimates of binary outcome data were calculated as the odds ratio (OR) and 95% confidence interval (CI). Continuous outcomes are reported as mean ± standard deviation (SD) or 95% CIs where appropriate. ORs were pooled with the random-effects model using the Mantel-Haenszel test. Meta-analyses performed included an assessment of heterogeneity of included studies through calculation of I^2^. Sensitivity analysis was performed for the primary outcome using studies that had no concerns in over 50% of quality assessment domains.

## Results

### Study selection

**Figure 2:**
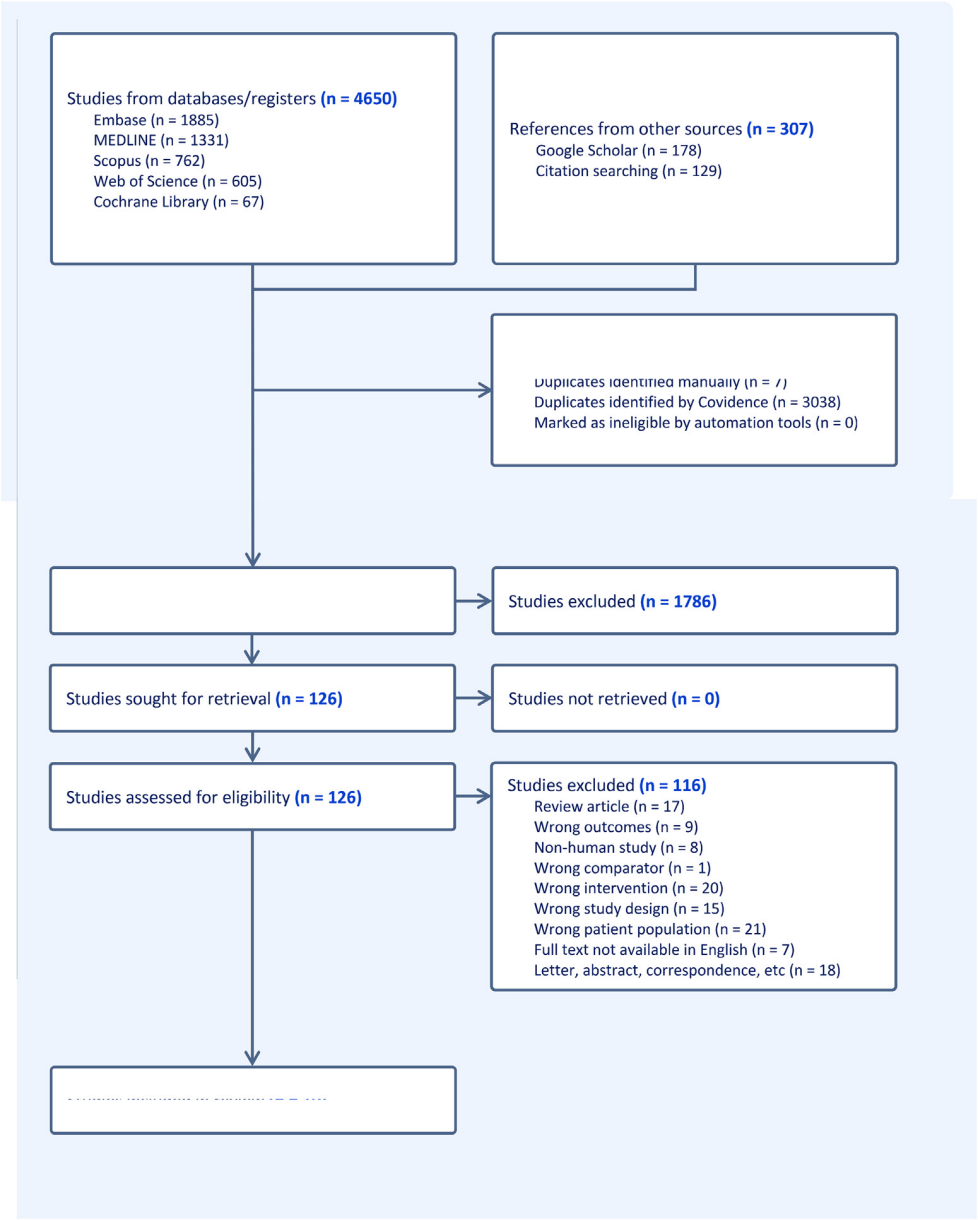
Preferred Reporting Items for Systematic Reviews and Meta-Analyses (PRISMA) flow diagram.

The systematic search identified a total of 4957 articles, of which 3045 were duplicates. Papers were initially screened by title and abstract, leaving 126 for full-text review. Overall, 10 papers met the inclusion and exclusion criteria (Figure 2) [Supplement 2].^3^^,^^12^^,^^16^^,^^20^, ^21^, ^22^, ^23^, ^24^, ^25^ The studies consisted of mostly case series,^3^^,^^12^^,^^16^^,^^20^^,^^21^^,^^23^^,^^24^^,^^26^ a case control study,^25^ and a randomised controlled trial.^22^ Two articles were based on the same data set and reported the same outcomes; hence, they were combined into one data source for this review.^3^^,^^24^

### Risk of bias in studies

The article by Fitoussi et al.^21^ was excluded based on the quality assessment, leaving nine data sources for this review (Supplement 3). The GRADE assessment is summarised in the summary of findings table (Supplement 4). Certainty of evidence ranged from ‘very low’ to ‘low’ due to the high number of observational studies.

### Study characteristics

The total sample size comprised 176 patients (range n=5-60 within included studies), with several patients undergoing bilateral breast reconstructions for a total 229 flaps. The type of simulated environment included both AR and VR (five and three, respectively). The technology used and techniques for perforator identification are summarised in Table 1. All papers studied DIEP-based reconstructions. Freidin et al.^16^ also included 4 TRAM flaps.

**Table 1:** Table showing type of simulated environment, technology and visualisation methods for included studies. VR = virtual reality, CTA – computed tomography angiogram, AR = augmented reality, NS = not specified, MRA = magnetic resonance angiogram, MS = Microsoft. * based on the same data set. ^†^ Excluded following quality assessment.

Study ID	Simulated environment type	Perforator imaging modality	Technology	Segmentation/reconstruction	Visualisation
Gómez-Cía^24^	VR	CTA	Desktop-based	VirSSPA	1:1 scale transparent template dressing
Gacto-Sánchez^3^	VR	CTA	Desktop-based	VirSSPA	1:1 scale transparent template dressing
Gacto-Sánchez^25^	VR	CTA	Desktop-based	VirSSPA	1:1 scale transparent template dressing
Hummelink^23^	AR	CTA	Projection mapping	Vitrea software	PicoPix PPX2480 Pico projector
Hummelink^22^	AR	CTA	Projection mapping	Vitrea software	PicoPix PPX2480 Pico projector
*Fitoussi*^21^	*AR*	*CTA*	*Desktop-based*	*AR software (NS)*	*Digital tablet (NS)*
Berger^20^	AR	MRA	Heads-up display	.	Magic Leap Version 1
Freidin^16^	VR	CTA	Heads-up display	D2P software	HTC Vive
Seth^12^	AR	CTA	Heads-up display	Synergy application	MS HoloLens 2
Necker^26^	AR	CTA	Heads-up display	MeshLab 2022	MS HoloLens 1

### Results of synthesis

**Figure 3:**
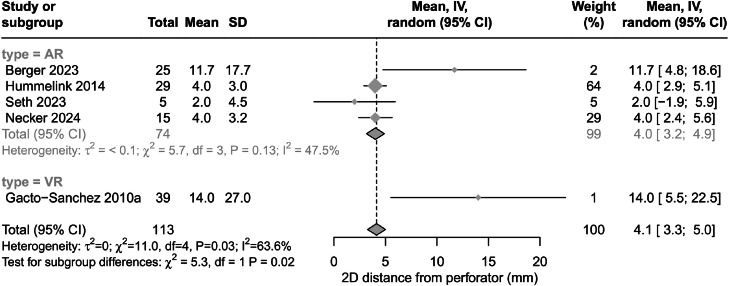
Forest plot diagram showing result of meta-analysis for outcome accuracy. Sub-analysis by simulated environment type. Results presented as distance from perforator in millimetres (mm). GRADE certainty of evidence was very low. Abbreviations: AR, augmented reality; CI, confidence interval; GRADE, Grading of Recommendations Assessment, Development and Evaluation; IV, instrumental variable; SD, standard deviation; VR, virtual reality.

Five articles reported the accuracy of perforator identification, described as a measure of 2D distance from perforator emergence through the rectus sheath and the skin marking.^3^^,^^12^^,^^20^^,^^23^^,^^24^^,^^26^ Overall, localisation of perforator emergence with AR and VR was 4.0 mm (95% CI 3.2-4.9) and 14.0 mm (95% CI 5.5-22.5), respectively, from the correct intraoperatively identified location (Figure 3). There was evidence of considerable heterogeneity between papers (I^2^=63.6%). After sensitivity analysis, random-effects model 2D distance increased slightly to 7.0 mm (95% CI 2.5-11.6) with the removal of one article by Seth et al.^12^ However, AR and VR accuracy remained comparable to pre-sensitivity analysis results (4.1 mm and 14.0 mm, respectively)

**Figure 4:**
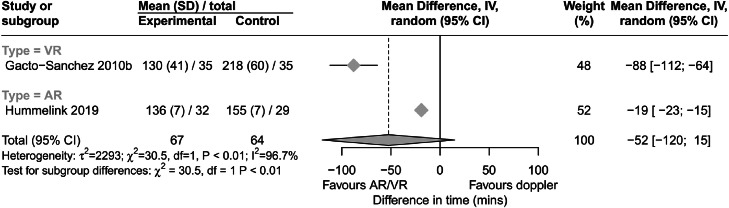
Forest plot diagram showing result of meta-analysis for outcome harvesting time. Sub-analysis by simulated environment type. Result presented as mean difference in time between simulated environment and Doppler (minutes). GRADE certainty of evidence was very low. Abbreviations: AR, augmented reality; CI, confidence interval; GRADE, Grading of Recommendations Assessment, Development and Evaluation; IV, instrumental variable; VR, virtual reality.

Two studies investigated the use of AR and VR on flap harvesting time compared to traditional unidirectional Doppler (Figure 4). Both found a significant reduction in harvesting time (88 min for VR and 19 min for AR).^22^^,^^25^ Processing time was the time required to transfer images to the simulated environment and segment and render them. The pooled mean image processing time was 39±47 min from four studies.^12^^,^^16^^,^^20^^,^^24^^,^^25^ Regarding total operating time, which was only reported in one study, preoperative VR was associated with shorter operative times than use of conventional Doppler ultrasound for perforator identification (478±56.94 vs 606.29 ± 81.94 min, P<0.05).^25^

**Figure 5:**
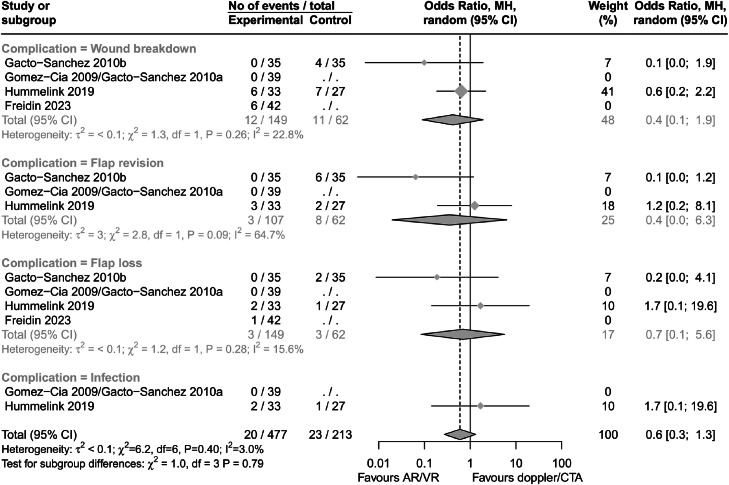
Forest plot diagram showing result of meta-analysis for outcome complications. Sub-analysis by complication type. Results presented as odds ratio. GRADE certainty of evidence was low. Abbreviations: AR, augmented reality; CTA, computed tomography angiography; GRADE, Grading of Recommendations Assessment, Development and Evaluation; MH, Mantel-Haenszel; VR, virtual reality.

Several papers reported complication data; however, single-arm case series could not be included in meta-analysis. Overall, use of extended reality environment for mapping perforators did not appear to significantly affect the risk of complications such as wound breakdown, flap revision, flap loss and infection (OR 0.6; 95% CI 0.3-1.3; P=0.20). Similarly, with individual complications, pooled results failed to meet significance (Figure 5). There was low heterogeneity between studies (I^2^=3.0%)

Berger et al. studied usability using the system usability scale (SUS), which reported an overall SUS of 67±10% (moderate to good) based on 10 cases from two consultants and one resident surgeon using AR technology. Scores were particularly high for questions 1 (‘I think that I would like to use this system frequently’, SUS=80±16%), 3 (‘I thought the system was easy to use’, SUS=75±16%) and 9 (‘I felt very confident using the system’, SUS=80±14%). However, response to ‘I think that I would need the support of a technical person to be able to use this system’ was also high (SUS=77±22%).^20^ Freidin et al. and Seth et al. reported the mean learning times for their AR heads-up display were 15 and 30 minutes, respectively.^12^^,^^16^

Financial outcomes were provided in three articles. Hummelink et al. described a €298 cost reduction per flap using AR, whereas Gacto-Sánchez et al. reported a large €1000 total savings per patient using VR.^22^^,^^25^ Although the costs of equipment and software licences were high, both papers reported that the main savings came from a reduction in surgery time. The cost of the MS HoloLens 2, used for heads-up display AR, was $3500 and did not include the additional costs of software licences.^12^

## Discussion

This review presents a synthesis of the available observational evidence relating to AR and VR technologies for perforator identification in breast reconstruction.^1^ The current literature relating to clinical use in oral and craniomaxillofacial surgery represents the largest evidence base supporting their practical application, defining benefits relating to virtual surgical planning and preoperative surgical simulation, real-time computer-assisted navigation improving intraoperative accuracy and reducing perioperative risk and offering aesthetic benefits and improved patient outcomes.^27^

In plastic and reconstructive surgery, the literature relating to use of extended reality technologies is widely limited at present to their educational applications. Quantitative evidence relating to their benefits and influence of surgical outcomes is limited in quantity and quality. This review aimed to determine the benefits of extended reality technologies in plastic surgery procedures in optimising perioperative planning and accurate and timely perforator identification to guide surgeons as to the value of introducing these technologies into their operative archetype.^15^^,^^28^

VR methodologies used included the VirSSPA tool, D2P software and desktop-based VR programs. These tools enable 3D reconstruction of body tissues from various imaging inputs, including CT, MR, positron emission tomography, single-photon emission computed tomography, ultrasound (US) and angiography, which aid the creation of 3D and 4D models that can be manipulated for surgical planning or 3D printing of anatomical models. Included VR technologies were typically desktop-based programs, with one study exporting inputs from the D2P software into a heads-up display. AR models included the Vitrea software and the Synergy application, software that can delineate structures from preoperative computed tomography angiography (CTA) scans including relevant vascular perforators and allow intraoperative visualisation of 3D images using projection hardware, including heads-up displays and projection mapping.

AR was superior to VR in intraoperative accuracy of perforator identification, with AR associated with localisation of perforator emergency an average of 4 mm from the correct intraoperative location. VR technologies were associated with an average 14-mm difference in localisation from the intraoperative findings. These findings suggest that AR is superior to VR in accuracy of perforator identification. Considering the wider context of existing imaging modalities, control US and 3D modelling within the included studies demonstrated a 7.92 ± 1.59 mm accuracy.^23^^,^^26^ This suggests that although AR technologies may offer improved accuracy, VR technologies may offer limited benefit compared to standard imaging techniques.

Existing meta-analysis demonstrated an average 6 mm accuracy with use of colour Doppler and MR angiography and mean 2.3-5 mm accuracy with use of computed tomography angiography (CTA), suggesting AR and VR offer little benefit in terms of accuracy based on the current data available in the academic literature.^29^ AR was suggested in one study to be more accurate (90.2%) with an increased sensitivity (97.4%) compared to Doppler (accuracy 82.4%, sensitivity 73.7%) in identifying perforators.^30^ Overall, inconsistencies in reported outcomes relating to accuracy of perforator identified with the assistance of extended reality technologies suggest that higher quality studies with large sample sizes are required before more definitive assessment of their potential use can be made. With perforator surgery, in which the size of perforators is typically <1 mm, only technologies that offer a high level of accuracy in perforator identification and are demonstrated to aid intraoperative correlation will have value compared to existing technologies. Further research directly comparing extended reality technologies to existing methodologies such as CT, MR and US imaging is required to provide reliable comparative analysis and further determine any potential benefits of extended reality imaging.^31^

In vitro and cadaveric studies have suggested AR technologies are associated with an average position error between 1-2 mm, suggesting the software itself offers an inherent high level of projection mapping accuracy that may be limited in the intraoperative environment by environmental factors including patient and staff movement, operator skill with equipment use or other factors that are more controlled in lab-based environments.^32^^,^^33^ The clinical data available and reported in this study demonstrate a significantly reduced benefit in improved accuracy of perforator identification compared to lab-based studies. This suggests that the logistical complexities and learning time required with intraoperative use may offer an increased layer of complexity to the course of perforator surgery and may not significantly aid accurate perforator identification. Notably, not all papers included data relevant to this outcome and of the four studies that did analyse intraoperative accuracy of perforator identification, significant heterogeneity in measurement of this outcome was found, relating to differences in technologies and visualisation techniques used.

Both studies assessing these outcomes suggested extended reality significantly reduced flap harvesting time, most notably with the use of VR at 88 minutes compared to unidirectional Doppler. Preoperative AR was also associated with a significant reduction in total operating time compared to Doppler US for perforator identification. No studies that compared the extended reality technologies to traditional CTA imaging, which is the gold standard for perforator identification, reported outcomes on reducing flap harvesting and total operating times.^34^ Although these results suggest that extended reality technologies offer benefit in reducing intraoperative times in free flap surgeries, the outcome data appear inconsistent with data suggesting extended reality technologies offer minimal benefit in improving accuracy of perforator identification, because without improved accuracy, it does not follow that flap harvesting time would be reduced. This suggests a level of observational or systematic bias in reporting of results or confounders introduced through methodological inconsistencies that limit reliability of this outcome data.

Complications reported in these studies, including wound breakdown, flap revision, failure and infection, were suggested to be reduced with use of stimulated environments. However, these outcomes failed to reach significance due to the available sample size from included studies and heterogeneity of outcome reporting. Further use and availability of temporal outcome data postoperatively will allow more comprehensive assessment of the influence of extended reality technologies on reducing complications associated with perforator-based flap reconstruction. In cases in which technologies did not significantly improve the accuracy of perforator identification, fewer operative complications may reduce operative times. These outcomes must be reproduced in further, higher quality observational studies.

A key consideration with introduction of extended reality technologies into the intraoperative environment is reports of usability and satisfaction from surgeons. Included studies noted that although systems were typically found easy to use and garnered a high rate of satisfaction from users, most noted that technological support was felt necessary for intraoperative use. Moreover, learning time for technologies was up to 30 minutes, in addition to processing time of approximately 40 minutes, adding a significant time consideration to effective intraoperative use. Although a reduction in learning time may occur with repeated use, frequent use would be required to gain familiarity, skill and mastery, which would ultimately result in meaningful time savings. Moreover, familiarity would have no effect on processing time, which may offer a practical limitation to widespread implementation of technologies unless further technological advancement reduces the overall processing time required.

The initial cost of extended reality systems is high, but the association of these technologies with significantly reduced surgical time and overall cost savings may outweigh the upfront financial commitment and the time associated with learning to use the software.

### Limitations

Limitations of this review include the low number of papers suitable for inclusion, reducing the sample size of the review cohort and the low quality of the available studies. The majority were retrospective case series, with only one higher quality randomised study. Studies with less than five participants were excluded due to the limitation of fewer participants on the quantitative validity of studies; this resulted in exclusion of several papers with relevant data for inclusion. Overall, these limitations reflect the low quality of papers with quantitative data relevant to the study outcomes currently available in the literature, highlighting the challenge of quantitative analysis of outcomes relating to use of extended reality technologies. Non-English language papers were excluded due to the lack of reliability of available translation services, which introduces language bias to the reported results.

## Conclusion

This review provides a systematic overview of the available literature on extended reality techniques for perforator mapping in free flap surgery for breast reconstruction. Extended reality, including AR and VR technologies, was associated with a significant reduction in both flap harvesting time and total operating time and the accuracy of perforator identification was comparable to existing imaging methodologies. The reliability of this outcome data is limited by the number and quality of available studies and methodological heterogeneity of available studies reduces the validity of interstudy comparison. Although data assessing the influence of extended reality technologies on reducing complications associated with perforator-based breast reconstruction are limited, further longitudinal studies involving the use of these technologies in the initial procedure may establish benefits relating to these outcomes. Further areas of beneficial research include larger scale quantitative analysis of individual technologies to allow inter-technology comparison with traditional imaging. As surgeons’ skill and familiarity with the use of extended reality equipment in the perioperative period improves, further evaluation of whether the pitfalls of equipment learning time and associated costs can be overcome. With both anecdotal evidence and further assessment of localisation accuracy, such technologies hold tangible benefit in aiding intraoperative perforator localisation and improving overall patient outcomes.

## Competing interests

The authors declare no conflicts of interest.

## Appendix Supplementary materials

Image, application 1

Image, application 2

Image, application 3

Image, application 4
