# Supplementary material for: Extended reality for mapping perforator-based flaps in breast reconstruction: a systematic review and meta-analysis
Source: JPRAS Open. 2025 Feb 27;44:269–83. doi: 10.1016/j.jpra.2025.02.011 (PMC12005224; doi:10.1016/j.jpra.2025.02.011)
Supplement: Supplementary file 1 [file mmc1.docx]

# **Search locations**

**Systematic review databases**

- Cochrane library

**Databases**

- MEDLINE
- EMBASE
- Global health
- Scopus
- Web of science

**Grey literature**

- Google scholar
- Abstracts/presentations

## Inclusion and exclusion criteria

| **Subject** | **Included** | **Excluded** |
| --- | --- | --- |
| *Populations* | Adults 18+ undergoing DIEP or thigh based flap breast reconstruction | Children |
| *Interventions* | Intraoperative use of augmented reality |  |
| *Comparison* | Normal intraoperative perforator identification |  |
| *Outcomes* | accuracy  harvesting time  operating time  complication rates (wound breakdown, flap revision, flap raise failure, flap loss, infection)  financial cost |  |
| *Study designs* | Primary research of any quantitative comparative study design from published and grey literature  Abstracts  Poster proceedings  RCT/observational | Non-scientific reports, letters and correspondences.  Reviews  Case reports  <5 patients  Single arm studies |
| *Language* | English | Any other language |
| *Publication dates* | Any date |  |

## Search strategy

| **Group 1** | **Group 2** |
| --- | --- |
| *Comparator* | *Population* |
| Augmented reality | Deep inferior epigastric perforator |
| AR | DIEP |
| Virtual reality | latissimus dorsi flap |
| VR | LD flap |
| Augmented modelling | Thoracodorsal artery perforator |
| Virtual modelling | TDAP |
| Holographic | TAP flap |
| Hololens | Lateral  Intercostal Artery Perforator |
|  | LICAP |
|  | intercostal artery perforators |
|  | ICAP |
|  | Transverse rectus abdominis muscle |
|  | TRAM |
|  | gluteal artery perforator |
|  | SGAP |
|  | IGAP |
|  | Lumbar Artery perforator |
|  | LAP |
|  | upper gracilis |
|  | TUG |
|  | DUG |
|  | VUG |
|  | profunda artery perforator |
|  | PAP |
|  | thigh perforator |
|  | LTP |
|  | ATP |

| Augmented reality | Perforator* |
| --- | --- |
| Virtual reality | Perforator flap* |
| Mixed reality | Breast recon* |
| Extended reality | Breast surgery |
| Holographic | Breast flap* |
| Hololens | Free flap* |
|  | Thigh flap* |
|  | Abdominal flap* |

# MEDLINE, via OVID

| **Term** | **Group** | **Search** | **Number** |
| --- | --- | --- | --- |
| 1 | Group 1 | Augmented reality.mp. or exp Augmented Reality/ or exp Surgery, Computer-Assisted/ | 47854 |
| 2 |  | Virtual reality.mp. or exp Virtual Reality/ | 22585 |
| 3 |  | Mixed reality.mp. | 1311 |
| 4 |  | Extended reality.mp. | 436 |
| 5 |  | Holographic.mp. | 8384 |
| 6 |  | Hololens.mp. | 459 |
| 7 |  | 1 or 2 or 3 or 4 or 5 or 6 | 77010 |
| 8 | Group 2 | exp Surgical Flaps/ or exp Perforator Flap/ or Perforator*.mp. | 74723 |
| 9 |  | Perforator flap*.mp. | 5757 |
| 10 |  | Breast recon*.mp. | 13265 |
| 11 |  | Breast surgery.mp. | 6456 |
| 12 |  | Breast flap*.mp. | 98 |
| 13 |  | Free flap*.mp. or exp Free Tissue Flaps/ | 16665 |
| 14 |  | Thigh flap*.mp. | 2175 |
| 15 |  | Abdominal flap*.mp. | 644 |
| 16 |  | 8 or 9 or 10 or 11 or 12 or 13 or 14 or 15 | 92252 |
| 17 | Combined | 7 and 16 | 688 |

# Embase, via OVID

| **Term** | **Group** | **Search** | **Number** |
| --- | --- | --- | --- |
| 1 | Group 1 | Augmented reality.mp. or exp computer assisted surgery/ or exp augmented reality/ | 51128 |
| 2 |  | exp virtual reality/ or Virtual reality.mp. | 38215 |
| 3 |  | Mixed reality.mp. | 1434 |
| 4 |  | Extended reality.mp. | 371 |
| 5 |  | Holographic.mp. | 5281 |
| 6 |  | Hololens.mp. | 714 |
| 7 |  | 1 or 2 or 3 or 4 or 5 or 6 | 92017 |
| 8 | Group 2 | Perforator*.mp. or exp inferior gluteal artery perforator flap/ or exp superior gluteal artery perforator flap/ or exp deep inferior epigastric perforator flap/ or exp perforator flap/ or exp thoracodorsal artery perforator flap/ | 13449 |
| 9 |  | Perforator flap*.mp. | 7904 |
| 10 |  | exp breast reconstruction/ or Breast recon*.mp. | 33264 |
| 11 |  | Breast surgery.mp. or exp breast surgery/ | 109298 |
| 12 |  | Breast flap*.mp. | 92 |
| 13 |  | exp free tissue graft/ or Free flap*.mp. | 23763 |
| 14 |  | exp anterolateral thigh flap/ or Thigh flap*.mp. | 3668 |
| 15 |  | exp surgical flaps/ or Abdominal flap*.mp. or exp tissue flap/ | 74939 |
| 16 |  | 8 or 9 or 10 or 11 or 12 or 13 or 14 or 15 | 183212 |
| 17 | Combined | 7 and 16 | 982 |

# Global Health, via OVID

| **Term** | **Group** | **Search** | **Number** |
| --- | --- | --- | --- |
| 1 | Group 1 | Augmented reality.mp. | 137 |
| 2 |  | Virtual reality.mp. | 640 |
| 3 |  | Holographic.mp. | 41 |
| 4 |  | Hololens.mp. | 5 |
| 5 |  | Mixed reality.mp. | 22 |
| 6 |  | Extended reality.mp. | 18 |
| 7 |  | 1 or 2 or 3 or 4 or 5 or 6 | 784 |
| 8 | Group 2 | Perforator*.mp. | 84 |
| 9 |  | Perforator flap*.mp. | 23 |
| 10 |  | Breast recon*.mp. | 182 |
| 11 |  | Breast surgery.mp. | 256 |
| 12 |  | Breast flap*.mp. | 2 |
| 13 |  | Free flap*.mp. | 152 |
| 14 |  | Thigh flap*.mp. | 31 |
| 15 |  | Abdominal flap*.mp. | 11 |
| 16 |  | 8 or 9 or 10 or 11 or 12 or 13 or 14 or 15 | 666 |
| 17 | Combined | 7 and 16 | 0 |

# Web of Science

| **Term** | **Group** | **Search** | **Number** |
| --- | --- | --- | --- |
| 1 | Group 1 | (((((ALL=(Augmented reality)) OR ALL=(Virtual reality)) OR ALL=(Mixed reality)) OR ALL=(Extended reality)) OR ALL=(Holographic)) OR ALL=(Hololens) | 188,423 |
| 2 | Group 2 | (((((((ALL=(Perforator*)) OR ALL=(Perforator flap*)) OR ALL=(Breast recon*)) OR ALL=(Breast surgery)) OR ALL=(Breast flap*)) OR ALL=(Free flap*)) OR ALL=(Thigh flap*)) OR ALL=(Abdominal flap*) | 260,031 |
| 3 | Combined | #1 AND #2 | 316 |

# Scopus

| **Term** | **Group** | **Search** | **Number** |
| --- | --- | --- | --- |
| 1 | Group 1 | ( TITLE-ABS-KEY ( augmented AND reality ) OR TITLE-ABS-KEY ( virtual AND reality ) OR TITLE-ABS-KEY ( mixed AND reality ) OR TITLE-ABS-KEY ( extended AND reality ) OR TITLE-ABS-KEY ( holographic ) OR TITLE-ABS-KEY ( hololens ) ) | 310,075 |
| 2 | Group 2 | ( TITLE-ABS-KEY ( perforator* ) OR TITLE-ABS-KEY ( perforator AND flap* ) OR TITLE-ABS-KEY ( breast AND recon* ) OR TITLE-ABS-KEY ( breast AND surgery ) OR TITLE-ABS-KEY ( breast AND flap* ) OR TITLE-ABS-KEY ( free AND flap* ) OR TITLE-ABS-KEY ( thigh AND flap* ) OR TITLE-ABS-KEY ( abdominal AND flap* ) ) | 174,493 |
| 3 | Combined | ( ( TITLE-ABS-KEY ( augmented AND reality ) OR TITLE-ABS-KEY ( virtual AND reality ) OR TITLE-ABS-KEY ( mixed AND reality ) OR TITLE-ABS-KEY ( extended AND reality ) OR TITLE-ABS-KEY ( holographic ) OR TITLE-ABS-KEY ( hololens ) ) ) AND ( ( TITLE-ABS-KEY ( perforator* ) OR TITLE-ABS-KEY ( perforator AND flap* ) OR TITLE-ABS-KEY ( breast AND recon* ) OR TITLE-ABS-KEY ( breast AND surgery ) OR TITLE-ABS-KEY ( breast AND flap* ) OR TITLE-ABS-KEY ( free AND flap* ) OR TITLE-ABS-KEY ( thigh AND flap* ) OR TITLE-ABS-KEY ( abdominal AND flap* ) ) ) | 394 |
