# Supplementary material for: Extended reality for mapping perforator-based flaps in breast reconstruction: a systematic review and meta-analysis
Source: JPRAS Open. 2025 Feb 27;44:269–83. doi: 10.1016/j.jpra.2025.02.011 (PMC12005224; doi:10.1016/j.jpra.2025.02.011)
Supplement: Supplementary file 2 [file mmc2.docx]

## Table of included studies

| **Study ID** | **Country** | **Funding** | **Study type** | **Duration (months)** | **Sample** | **Age** | **BMI** | **Flap type** | **Type** | **Technology** | **Outcome(s)** |
| --- | --- | --- | --- | --- | --- | --- | --- | --- | --- | --- | --- |
| Gomez-Cia 2009* | Spain | Andalusian  Department of Health | Case series | 6 | 12 | NR | NR | DIEP | VR | **Desktop-based virtual reality** CTA, VirSSPA (3D image recon software) 1:1 scale transparent template dressing | Accuracy,  Processing time,  Complications |
| Gacto-Sanchez 2010b | Spain | Andalusian  Department of Health | Case-control | 13 | 35 | 48.89 ± 7.52 | 27.17 ± 3.66 | DIEP | VR | **Desktop-based virtual reality** CTA, VirSSPA (3D image recon software) 1:1 scale transparent template dressing Second arm: Doppler | Harvesting time,  Operating time,  Processing time,  Complications,  Financial |
| Gacto-Sanchez 2010a* | Spain | Andalusian  Department of Health | Case series | 5 | 12 | NR | NR | DIEP | VR | **Desktop-based virtual reality** CTA, VirSSPA (3D image recon software) 1:1 scale transparent template dressing | Accuracy,  Complications |
| Hummelink 2014 | The Netherlands | None | Case series | 3 | 9 | NR | NR | DIEP | AR | **Projection mapping augmented reality** CTA, unidirectional doppler Vitrea software (CT scan) PicoPix PPX2480 Pico projector | Accuracy |
| Hummelink 2019 | The Netherlands | Dutch government | Randomised controlled trial | NR | 60 | AR: 52+9,  D: 50+8 | AR: 26.5+2,  D: 26.8+2.7 | DIEP | AR | **Projection mapping augmented reality** CTA, VitreaAdvanced fX Workstation PicoPix PPX2480 Pico projector Second arm: Doppler | Harvesting time,  Complications,  Financial |
| Fitoussi 2021† | France | None | Case series | NR | 12 | NR | NR | DIEP | AR | **Desktop-based virtual reality** CTA, Segemntation, CAD, "AR" software (VR) 1:1 scale transparent template dressing | Accuracy,  Complications |
| Berger 2023 | Austria | Grant - Austrian  Research Society FFG | Case series | 29 | 10 | 46.6 | 28.4 | DIEP | AR | **Head up display augmented reality** MRI/MRA, 3D surface scanning algorithm, Doppler US Magic Leap Version 1 (AR visualisaition software) | Accuracy,  Ease of use,  Processing time,  Complications |
| Freidin 2023 | Palastine | None | Case series | 21 | 30 | NR | NR | DIEP,  TRAM | VR | **Heads up display virtual reality** CTA, D2P software (segmentation) HTC Vive system | Processing time,  Complications,  Ease of use |
| Seth 2023 | Australia, Denmark | None | Case series | 8 | 5 | NR | NR | DIEP | AR | **Head up display AR** CTA, Materialise Mimics Innovation Suite (segmentation) Blender, Synergy application on MS HoloLens 2 | Accuracy,  Processing time,  Ease of use,  Financial |
| Necker 2024 | USA | Bavaria California Technology Center | Case series | NR | 15 | 48.6± 9.5 | 27.3±  3.6 | DIEP | AR | **Head up display AR**  CTA, 3D segmentation in MeshLab 2022  HoloDIEP application on MS HoloLens 1 | Accuracy |
