# Supplementary figures and images for: Extended reality for mapping perforator-based flaps in breast reconstruction: a systematic review and meta-analysis
Source: JPRAS Open. 2025 Feb 27;44:269–83. doi: 10.1016/j.jpra.2025.02.011 (PMC12005224; doi:10.1016/j.jpra.2025.02.011)

## Results of quality assessment using JBI checklist


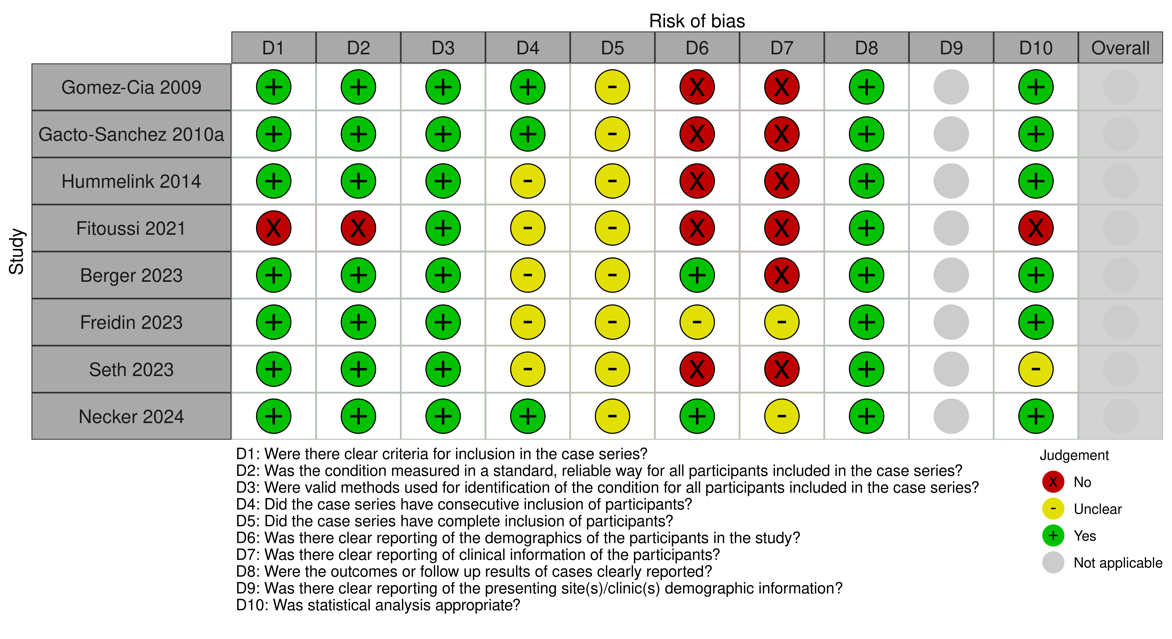

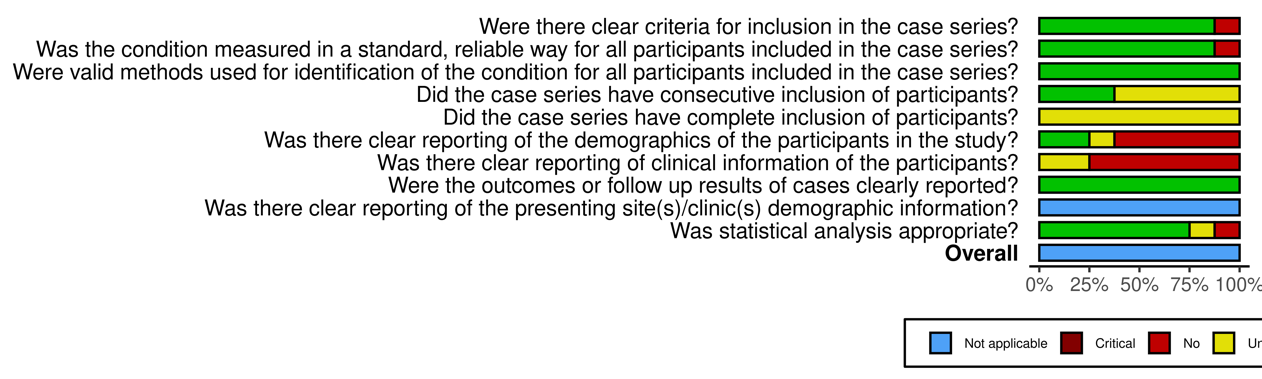


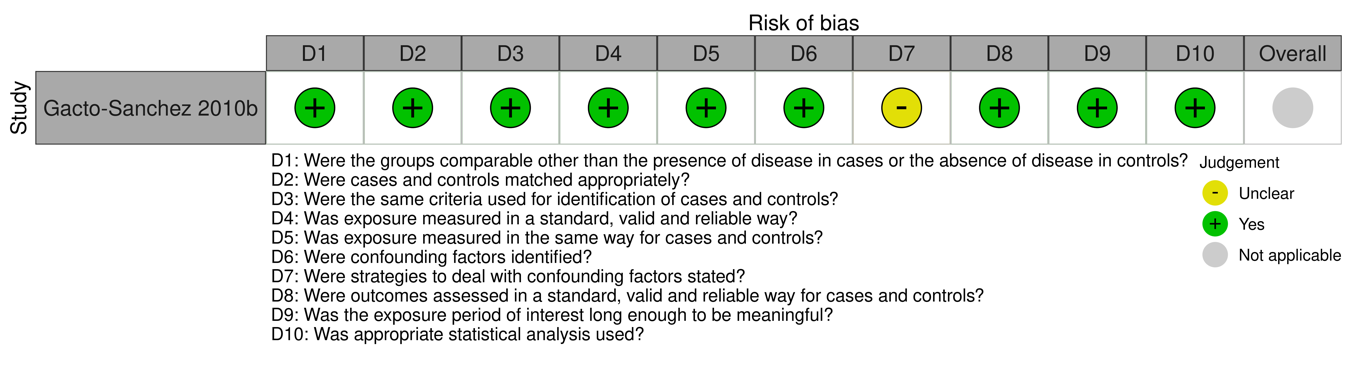

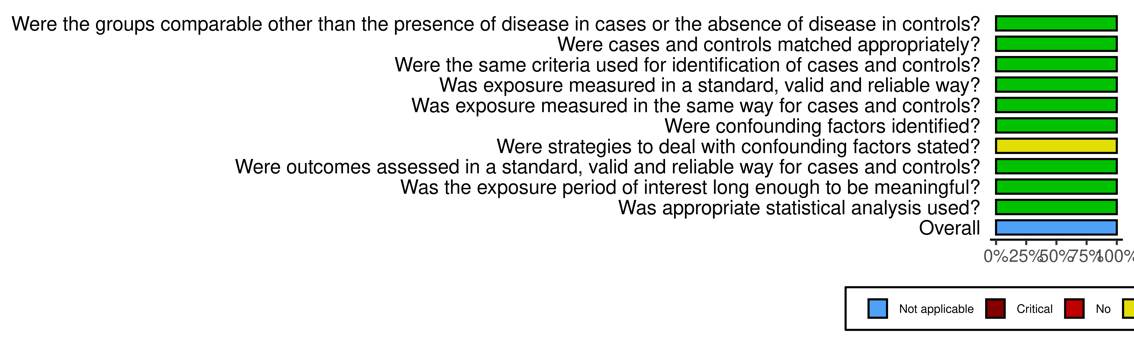

Supplement: Supplementary file 3 [file mmc3.docx]

## GRADE Summary of Findings Table


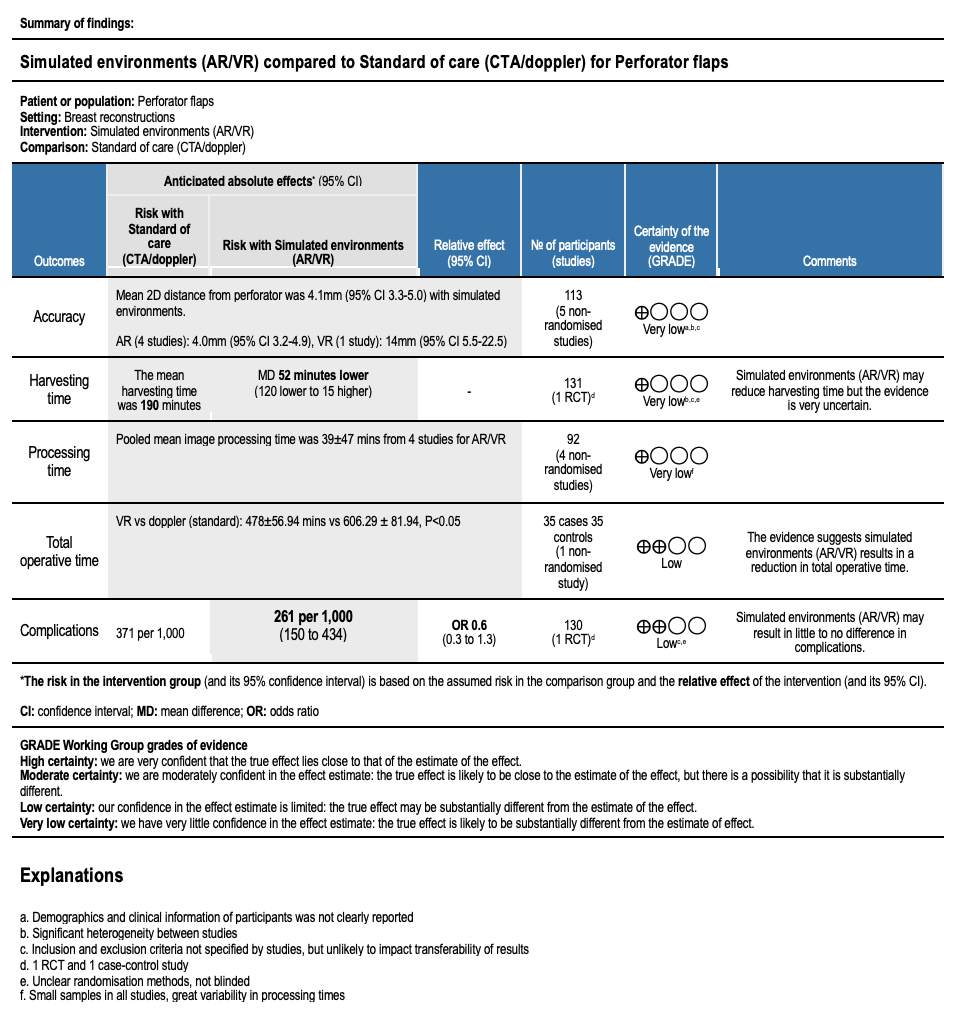

Supplement: Supplementary file 4 [file mmc4.docx]
